# Dealing with mpox in southeast Asia: strategy, not panic

**DOI:** 10.1016/j.lansea.2024.100475

**Published:** 2024-09-10

**Authors:** The Lancet Regional Health — Southeast Asia

Mpox, an infectious disease caused by monkeypox virus, is endemic to central and west Africa. Mpox was previously declared a public health emergency of international concern (PHEIC) in July 2022. The PHEIC status of mpox was removed in May 2023 after the number of cases waned. However, the 15,600 cases and 537 deaths reported in 2024 (until August) surpassed the counts of 2023. On Aug 14, 2024, Dr Tedros Adhanom Ghebreyesus, WHO Director-General, once again declared mpox a PHEIC. Should countries in southeast Asia be worried about this second announcement?

There are two clades (groups) of mpox: clade I (prevalent in central Africa) and clade II (prevalent in west Africa). Just 1 day after WHO's second declaration, the first case of mpox was reported from Sweden. This is the first time an mpox case of clade I was reported outside Africa (clade Ib). Considering that the estimated case fatality of clade I is higher (10%) than clade II (1–3%), there could be a major risk to global health. Also, preliminary results of a recent clinical trial showed that the antiviral tecovirimat is not effective against clade Ib. The mpox vaccine Jynneos is mainly used in individuals aged 18 years and older. Most of the deaths from the Democratic Republic of the Congo were reported in children younger than 15 years, most probably due to inadequate nutrition and healthcare. Outside Africa, other than Sweden, Pakistan has registered four mpox cases and one case has been reported from Thailand (as of Aug 21, 2024). It is yet to be confirmed whether the mpox cases in Pakistan and Thailand are of clade I or not.

In our Editorial published in July 2023, we emphasised that over-preparedness is better (with respect to future deadly variants of SARS-CoV-2). The 2022 outbreak of mpox led to low-income and middle-income countries in southeast Asia preparing themselves for an outbreak. At present, several countries have increased screening for fever and mpox symptoms at entry points such as seaports and airports. High-level meetings are separately being held in India and Pakistan on the advice of the prime ministers to review their countries' preparedness for a new mpox outbreak. In India, the latest case of mpox was in March 2024 and there is no active case as of Aug 22, 2024. Around 32 laboratories across India have been assigned to test mpox. Bangladesh's Directorate General of Health Services has started a hotline for mpox reporting. In Indonesia, 88 mpox cases were confirmed (as of Aug 17, 2024) of which 87 recovered and 54 were of clade IIb. Around 12 national reference laboratories in Indonesia have been assigned for diagnosis and whole-genome sequencing.

Some experts in India have criticised WHO's declaration for creating unnecessary panic and urged the focus to be on increasing awareness among high-risk individuals including people living with HIV. As monkeypox virus is a close relative of smallpox virus, the experts believe that individuals (older than 44 years) who received first generation smallpox vaccine during early vaccination programmes would possibly be immune to mpox. Several studies point out that cellular and humoral immunity against vaccinia virus imparted by first generation smallpox vaccines are long-lived. However, more studies testing the effectiveness of mpox vaccines are needed. Panic in the media can be avoided through timely actions of health ministries. For example, the State of Kerala in India, which bagged the top rank for the fourth consecutive year regarding progress towards the Sustainable Development Goals, was the first Indian state to report COVID-19 and mpox cases. Rather than creating panic and negatively impacting people's livelihoods, the government of Kerala focused on active surveillance, thereby reassuring the public.

Regarding mpox prevention, WHO has called for ‘targeted vaccinations’ instead of mass vaccinations. However, like in the COVID-19 pandemic, high-income countries—which have the fewest mpox cases—are ‘hoarding’ excessive quantities of the vaccine. WHO must ensure creation of a treaty for resolving the current vaccine inequity. In southeast Asia, the mpox vaccine Jynneos is not readily available in many countries. The vaccine production facilities in India could be used to manufacture mpox vaccines and possibly solve the vaccine inequity prevalent in southeast Asia and Africa. The Serum Institute of India is currently working on mpox vaccine development. The Technical Report on Mpox in Indonesia 2023 released in April 2024 recommended increasing active and passive surveillance, and strengthening laboratory networks. Health authorities must promote public awareness to prevent stigma associated with high-risk individuals. Regarding future research on mpox in southeast Asia, requirement of Bio Safety Level 2 laboratories with Bio Safety Level 3 practices is a major challenge. As climate change might lead to redistribution of animal hosts, surveillance using the One Health approach is required, and would prevent rare recombination events between clades I and II.

It is worrisome that even after 2 years of political negotiations, the WHO member states have not been able to bring out the world's first Pandemic Treaty. There are different hypotheses suggesting that declaration of PHEIC is political. Interestingly, mpox was not declared a PHEIC when African countries like Uganda, Rwanda, Kenya, and Burundi reported mpox cases for the first time in July 2024. WHO's second declaration of PHEIC came just a day after the Africa Centres for Disease Control and Prevention declared mpox as a public health emergency of continental security (PHECS). The cited reasons were to avoid global complacency or panic. But it would be clear injustice if mpox was to lose its PHEIC status once the cases wane in high-income countries—especially in the western part of the globe. *The Lancet Regional Health – Southeast Asia* calls for risk assessment for different countries and equal representation while deciding whether to declare a PHEIC. Let us prepare for the worst (strategy) and hope for the best (not panic).

